# Erfassung von Medikationsfehlern in Deutschland – ein Workshopbericht

**DOI:** 10.1007/s00103-025-04175-6

**Published:** 2025-12-17

**Authors:** Birgit Vogt, Claudia Kayser, Ursula Köberle, Hugo Kupferschmidt, Claudia Langebrake, Beate Müller, Wolfgang Rascher, André Said, Oliver Schwalbe, Petra Thürmann

**Affiliations:** 1 Bundesärztekammer, Wissenschaftliches Sekretariat Aktionsplan AMTS, AkdÄ, Berlin, Deutschland; 2 Abteilung 5 – Forschung, Bundesinstitut für Arzneimittel und Medizinprodukte, Bonn, Deutschland; 3 Bern, Schweiz; 4 Charité – Universitätsmedizin Berlin, Berlin, Deutschland; 5 Klinikapotheke und Klinik für Stammzelltransplantation, Universitätsklinikum Hamburg-Eppendorf, Hamburg, Deutschland; 6 Ausschuss für Pharmazeutische Interventionen, Bundesverband Deutscher Krankenhausapotheker (ADKA e. V.), Berlin, Deutschland; 7 Institut für Allgemeinmedizin, Medizinische Fakultät und Uniklinik Köln, Universität zu Köln, Köln, Deutschland; 8 Kinder- und Jugendklinik, Erlangen, Deutschland; 9 Arzneimittelkommission der Deutschen Apotheker (AMK), Berlin, Deutschland; 10 WIVA – Wissenschaftliches Institut der Apothekerkammer Westfalen-Lippe für Versorgungsforschung in der Apotheke, Münster, Deutschland; 11 Lehrstuhl für Klinische Pharmakologie, Fakultät für Gesundheit, Universität Witten/Herdecke, Helios Universitätsklinikum Wuppertal, Witten, Deutschland; 12 Lehrstuhl für Klinische Pharmakologie, Fakultät für Gesundheit, Department Humanmedizin, Universität Witten/Herdecke, Helios Universitätsklinikum Wuppertal, Heusnerstr. 40, 42283 Wuppertal, Deutschland

## Hintergrund zur Erfassung von Nebenwirkungen und Medikationsfehlern

Die Anwendung von Arzneimitteln ist eine sehr wichtige und häufige Maßnahme zur Prävention, Linderung oder Heilung von Beschwerden und Erkrankungen. In Deutschland sind über 100.000 Fertigarzneimittel zugelassen, etwa die Hälfte davon ist verschreibungspflichtig [1]. Zusätzlich wurden im Bereich des Paul-Ehrlich-Instituts (PEI) mehr als 2800 biomedizinische Arzneimittel zugelassen [2]. Im Jahr 2023 belief sich das Volumen verordneter Arzneimittel im Rahmen der gesetzlichen Krankenversicherung (GKV) auf insgesamt 48,4 Mrd. definierte Tagesdosen (DDD; [3]). 2023 wurden in Apotheken zusätzlich ca. 480 Mio. Packungen im Rahmen der Selbstmedikation an Patientinnen und Patienten abgegeben [4]. Die mit der Medikation verbundenen Risiken, wie etwa Nebenwirkungen von Arzneimitteln, werden im Rahmen der ärztlichen Behandlung sorgfältig abgewogen und fließen in die gemeinsam mit den Patientinnen und Patienten zu treffende Therapieentscheidung ein [5]. Ergänzend erfolgen in der Apotheke eine adäquate Information und Beratung der Patientinnen und Patienten zur Arzneimitteltherapie.

Der Begriff „Nebenwirkungen“ wird im deutschsprachigen Raum synonym mit der Bezeichnung „Unerwünschte Arzneimittelwirkungen“ (UAW) verwendet und darunter werden schädliche und unbeabsichtigte Reaktionen auf Arzneimittel verstanden (s. Infobox). Nebenwirkungen von Arzneimitteln können sowohl bei bestimmungsgemäßem Gebrauch im Rahmen der ärztlich verordneten Therapie als auch in der Selbstmedikation auftreten. Dies gilt auch bei sorgfältiger Indikationsstellung sowie unter Berücksichtigung von Kontraindikationen, Interaktionen und der korrekten Dosierung [6, 7].

Ebenso können Nebenwirkungen von Arzneimitteln auch durch Medikationsfehler entstehen. Diese sind grundsätzlich vermeidbar und unbeabsichtigt, aber führen nicht zwangsläufig zu schädlichen Wirkungen oder dem Versagen der Arzneimitteltherapie (s. Infobox). Medikationsfehler können vielfältige Ursachen haben. So spielen unter anderem eine unzweckmäßige Auswahl, Anwendung oder Dosierung des Arzneimittels eine Rolle, ebenso wie Probleme bei der Verordnung, Abgabe oder Kommunikation. Verwechslungen von Arzneimitteln, etwa aufgrund ähnlich klingender Arzneimittelbezeichnungen oder ähnlicher Verpackungen – sogenannte Look-alikes und Sound-alikes (LASA) – sind ebenfalls ein häufiges Problem und können zu Medikationsfehlern führen [8]. Darüber hinaus können Medikationsfehler durch nicht beachtete Wechselwirkungen zwischen Arzneimitteln, Lebensmitteln oder Nahrungsergänzungsmitteln entstehen. Um eine sichere Arzneimitteltherapie zu gewährleisten, muss der gesamte Medikationsprozess mit allen Beteiligten betrachtet werden: Ärztinnen und Ärzte, Apothekerinnen und Apotheker, Pflegefachpersonen, Fachkräfte weiterer Gesundheitsberufe, Patientinnen und Patienten sowie deren Angehörige oder beteiligte Dritte [7]. Die Verhinderung von Medikationsfehlern muss im Kontext eines optimalen Medikationsprozesses gesehen werden und ist daher ein zentraler Bestandteil der Arzneimitteltherapiesicherheit (AMTS; s. Infobox).

### Epidemiologie und Relevanz von Nebenwirkungen

Nebenwirkungen von Arzneimitteln sind häufige Ursachen für Notfallbehandlungen und stationäre Aufnahmen. Die internationale Studienlage zeigt, dass etwa 5–10 % aller Krankenhauseinweisungen auf das Auftreten von Nebenwirkungen zurückzuführen sind [9–11]. Eine wesentliche Rolle nehmen dabei vermeidbare Medikationsfehler ein [12–14]. Auch in Deutschland sind Arzneimittelnebenwirkungen ein relevanter Grund für stationäre Aufenthalte und ein beträchtlicher Teil könnte verhindert werden [15, 16]. Hierzulande sind Nebenwirkungen von Arzneimitteln für etwa 3–7 % der Notaufnahmen im Krankenhaus verantwortlich [17–20]. Auf Basis internationaler Untersuchungen, einschließlich Deutschlands, kann davon ausgegangen werden, dass innerhalb der Bundesrepublik jährlich etwa 250.000 Krankenhauseinweisungen mit vermeidbaren Medikationsfehlern in Verbindung stehen [21]. Im deutschen Gesundheitswesen führt das Auftreten von Nebenwirkungen durch Arzneimittel – je nach gesundheitsökonomischen Kennzahlen – zu einer jährlichen finanziellen Belastung zwischen 87 Mio. und über einer Milliarde Euro [15, 18, 22].

Ältere und hochbetagte Menschen sind häufig von Multimorbidität betroffen und werden mit mehreren Arzneimitteln gleichzeitig behandelt, was als Polymedikation bezeichnet wird [23, 24]. Polymedikation ist grundsätzlich mit einem höheren Risiko für Probleme in der Arzneimitteltherapie verbunden [25]. Nebenwirkungen von Arzneimitteln treten insbesondere bei älteren Menschen auf und führen bei ihnen besonders häufig zu Krankenhausaufenthalten [26, 27].

Auch bei Kindern sind etwa 3–10 % der Aufnahmen ins Krankenhaus auf Nebenwirkungen von Arzneimitteln zurückzuführen, die zum Teil aufgrund vermeidbarer Medikationsfehler beobachtet werden [28–30]. Die clusterrandomisierte Studie „Verbesserung der Versorgung von Kindern und Jugendlichen mit Arzneimitteln durch Erhöhung der Arzneimitteltherapiesicherheit“ (KiDSafe) analysierte beispielsweise fast 42.000 ungeplante stationäre Aufnahmen und identifizierte dabei einen arzneimittelbedingten Anteil von 4,1 % [31].

### Erfassung von Nebenwirkungen und Medikationsfehlern

Eine wesentliche Quelle von Verdachtsfällen von Nebenwirkungen stellen die sogenannten Spontanmeldungen dar [32]. Angehörige des Arzt- bzw. Apothekerberufes sind gemäß ihren jeweiligen Berufsordnungen verpflichtet, Berichte über vermutete Nebenwirkungen bzw. Arzneimittelrisiken zu melden. Diese Berichte werden an die entsprechenden Arzneimittelkommissionen der Kammern der Heilberufe – die Arzneimittelkommission der deutschen Ärzteschaft (AkdÄ) bzw. die Arzneimittelkommission der Deutschen Apotheker (AMK) – übermittelt [33, 34]. Auch Inhaber der jeweiligen Arzneimittelzulassung sind verpflichtet, Verdachtsfälle von Nebenwirkungen entgegenzunehmen, zu dokumentieren und wie die Arzneimittelkommissionen an die zuständige Bundesoberbehörde (BOB) – das Bundesinstitut für Arzneimittel und Medizinprodukte (BfArM) oder das Paul-Ehrlich-Institut (PEI) – elektronisch weiterzuleiten. Die Spontanmeldungen können mithilfe statistischer Verfahren analysiert werden und bilden zusammen mit pharmakoepidemiologischen Untersuchungen die Grundlage der Pharmakovigilanz [35]. Aus der Analyse und Bewertung auftretender Risikosignale können risikominimierende Maßnahmen abgeleitet werden, wie etwa Warnhinweise, Einschränkungen der Indikationsgebiete oder sogar der Widerruf der Zulassung.

Daneben gibt es für Berichte über Medikationsfehler, unabhängig davon, ob sie gesundheitliche Folgen für die Patientinnen und Patienten haben, weitere Möglichkeiten zur Dokumentation. So haben sich Fehlermeldeportale verschiedener Institutionen etabliert, die häufig nicht nur Medikationsfehler, sondern auch andere Fehler erfassen. Die Portale richten sich teilweise an unterschiedliche Zielgruppen und setzen jeweils einen anderen Fokus. Exemplarisch seien an dieser Stelle das Fehlerberichts- und Lernsystem für Hausarztpraxen „Jeder-Fehler-zählt“ (JFZ), das Lern- und Berichtssystem „Critical-Incident-Reporting-System Nordrhein-Westfalen“ (CIRS-NRW) und das „Dokumentationssystem für pharmazeutische Interventionen im Krankenhaus sowie Medikationsfehler“ (DokuPIK) des Bundesverbands Deutscher Krankenhausapotheker e. V. (ADKA) aufgeführt. Auch in Giftinformationszentren der Länder (GIZ) machen Anfragen mit Arzneimittelbezug einen Anteil von 30–40 % der Beratungsfälle aus. Ebenso sind Fehler bei der Arzneimitteltherapie bei den Gutachterkommissionen und Schlichtungsstellen regelmäßig Gegenstand der Konsultationen [36].

Die AkdÄ führte von 2015 bis 2017 ein Forschungsprojekt zur „Erfassung und Bewertung von Medikationsfehlern“ durch [37]. Dieses Projekt wurde vom Bundesministerium für Gesundheit (BMG) gefördert. Ziel war es, herauszufinden, ob die Erfassung von Medikationsfehlern innerhalb der bestehenden Strukturen des Spontanmeldesystems möglich ist. Die Analyse bestätigte diese Möglichkeit. Zusätzlich wurde festgestellt, dass Medikationsfehler in Deutschland an verschiedenen Stellen erfasst und ausgewertet werden, jedoch bisher keine umfassende Zusammenführung dieser Daten existiert, was sicherlich zu einem Erkenntnisgewinn führen würde.

In Anknüpfung an das AkdÄ-Projekt und zum besseren Verständnis der jeweiligen Erfassungssysteme sowie Ausloten gemeinsamer bzw. sich ergänzender Auswertungen wurde im Rahmen des Aktionsplans AMTS 2021–2024 ein Workshop durchgeführt [38, 39]. Expertinnen und Experten verschiedener Projekte, Initiativen und Institutionen beleuchteten den Umgang mit Berichten über Medikationsfehler, die ihnen übermittelt oder im Rahmen ihrer Tätigkeit gesammelt und ausgewertet wurden.

Dieser Workshopbericht benennt zunächst die Aspekte zu Medikationsfehlern, die in der Expertendiskussion im Rahmen des Workshops sowie in einer ergänzenden Erhebung von Informationen erfasst wurden. Im Anschluss wird ein Überblick über die verschiedenen Projekte, Initiativen und Institutionen sowie ihre Rolle im Melde- und Berichtswesen zu Medikationsfehlern in Deutschland gegeben. Danach werden die Ergebnisse des Workshops und der Erhebung vorgestellt, insbesondere zu Anzahl und Arten von Medikationsfehlern, den Settings und betroffenen Arzneimittelgruppen, die aus den gesammelten Berichten hervorgehen. Herausforderungen und förderliche Faktoren im Meldewesen werden aufgezeigt. Abschließend werden das Zusammenwirken der Beteiligten und die Einrichtung von Schnittstellen thematisiert, bevor die Ergebnisse eingeordnet und Schlussfolgerungen gezogen werden.

## Workshop und ergänzende Erhebung zu Medikationsfehlern

### Expertendiskussion im Workshop.

Zur Teilnahme am „Workshop zur Verbesserung der zentralen Erfassung von Medikationsfehlern“ im Mai 2022 wurden Expertinnen und Experten der zuständigen Behörden, Arzneimittelkommissionen und Institutionen sowie etablierter Fehlermeldeprojekte angesprochen [39]. Der Workshop fand ohne die Teilnahme der Vertreterinnen und Vertreter des PEI statt. Eine Übersicht der teilnehmenden Institutionen ist Tab. 1 zu entnehmen. Die Mitglieder der Koordinierungsgruppe AMTS konnten ebenfalls am Workshop teilnehmen und waren zum Teil vor Ort oder online dabei [40]. Insgesamt nahmen 26 Personen an dem Workshop teil. Anhand von Impulsreferaten und Projektvorstellungen wurden relevante Aspekte mittels moderierter Diskussion im Expertenkreis erörtert. Wesentliche Gesichtspunkte in der Diskussion waren:

- Zielsetzung der Meldeform,
- Art der Fehlermeldungen,
- Erfassung und ggf. Weiterleitung der Berichte,
- Herausforderungen bei der Erfassung,
- mögliche Meldehemmnisse und Fehlerkultur,
- Einrichtung möglicher Schnittstellen für den Informationsaustausch mit den Arzneimittelkommissionen oder Behörden,
- Einleitung regulatorischer Gegenmaßnahmen (risikominimierende Maßnahmen) durch die Behörden.

**Tab. 1:** Am Workshop und an der Erhebung teilnehmende Institutionen.

Projekte und Initiativen	Meldesystem der Kinder- und Jugendklinik des Universitätsklinikums Erlangen ([41]; MF in der Pädiatrie)
	„Jeder-Fehler-zählt“ (JFZ), Fehlerberichts- und Lernsystem für Hausarztpraxen (https://jeder-fehler-zaehlt.de/)
	„Critical-Incident-Reporting-System Nordrhein-Westfalen“ (CIRS-NRW) (https://www.cirsmedical.de/nrw/)
	„Dokumentation Pharmazeutischer Interventionen im Krankenhaus“ (ADKA-DokuPIK) (https://aminfo.adka.de/adka-datenbanken/adka-dokupik-2)
Institutionen, Arzneimittelkommissionen, Behörden	Giftinformationszentren der Länder (GIZ)
	Arzneimittelkommission der Deutschen Apotheker (AMK) (www.arzneimittelkommission.de)
	Arzneimittelkommission der deutschen Ärzteschaft (AkdÄ) (https://www.akdae.de/)
	Bundesinstitut für Arzneimittel und Medizinprodukte (BfArM) (https://www.bfarm.de/DE/Home/_node.html)

Das Ergebnis des Workshops wurde protokolliert und auf der Homepage der AkdÄ veröffentlicht [39].

### Ergänzende Erhebung.

Aufbauend auf den Workshopergebnissen wurden in einem weiteren Schritt alle Expertinnen und Experten der insgesamt 8 einbezogenen Projekte, Initiativen, Institutionen, Arzneimittelkommissionen und Behörden gebeten, weitere spezifische Informationen zusammenzustellen. Alle 8 Expertinnen und Experten füllten die vorstrukturierten Tabellen zur Charakterisierung der einzelnen Stellen aus, die im E‑Mail-Verfahren übermittelt wurden. Es lagen somit insgesamt 8 Rückmeldungen zur Auswertung folgender Aspekte vor:

- Zielsetzung des Projekts bzw. Aufgabe der Institution im Bereich Medikationsfehler,
- Zielgruppe für den Meldeweg und Reichweite,
- Link zur Homepage bzw. zum Meldeportal,
- Anzahl gemeldeter Medikationsfehler pro Jahr,
- häufig betroffene Arzneimittel(gruppen),
- Art und Setting relevanter Medikationsfehler,
- Umgang mit Berichten über Medikationsfehler,
- Herausforderungen bei der Erfassung, z. B. Meldehemmnisse oder Fehlerkultur,
- Zusammenarbeit mit weiteren Akteurinnen und Akteuren, z. B. Einrichtung von Schnittstellen ermöglicht bzw. denkbar,
- weitere Aspekte und Sonstiges, z. B. Verweis auf veröffentlichte Untersuchungen bzw. Forschungsergebnisse, Konsequenzen aus den Erkenntnissen, Etablierung von „Awareness-Wochen“, Veröffentlichungen in Fachmedien.

Die Teilnehmenden konnten die Antworten als Freitext formulieren. Die Rückmeldungen wurden im Wissenschaftlichen Sekretariat des Aktionsplans AMTS ausgewertet und zusammengefasst.

## Ergebnisse

### Stellen zur Erfassung von Medikationsfehlern in Deutschland

#### Spontanmeldewesen und GIZ.

Die zuständigen Bundesoberbehörden BfArM und PEI sowie die Arzneimittelkommissionen der Kammern der Heilberufe – AkdÄ und AMK – erfüllen arzneimittelrechtlich verankerte Aufgaben im Spontanmeldewesen bzw. bei der zentralen Erfassung von Nebenwirkungen, inkl. Medikationsfehler (§§ 62–63 Arzneimittelgesetz – AMG). Sie erfassen und bewerten die Berichte über Medikationsfehler strukturiert innerhalb der nationalen Pharmakovigilanzstruktur, der Fokus liegt hier auf der Sicherheit des Arzneimittels und seiner Anwendung. Auch die GIZ der Länder haben bestimmte chemikalienrechtlich verankerte Aufgaben. Jedoch liegt der Fokus hier auf der stoffbezogenen Beratung in akuten Vergiftungssituationen, zu einem erheblichen Teil auch im Zusammenhang mit Arzneimitteln. Die GIZ leiten Berichte über Medikationsfehler, z. B. versehentliche Überdosierungen, *nicht* systematisch an die zuständigen Bundesoberbehörden weiter.

#### Systeme im Rahmen des Risikomanagements.

Davon abzugrenzen sind solche Stellen, die etwa im Rahmen des Risikomanagements entsprechende Berichts‑, Dokumentations- und Lernsysteme eingerichtet haben, um unter anderem die Patientensicherheit zu stärken. Diese Systeme haben teilweise eher die Prozesskette im Fokus, beispielsweise Informationslücken bzgl. Medikation beim Sektorenübergang von Patientinnen und Patienten. Institutionell verankert sind beispielsweise die Initiativen „Jeder-Fehler-Zählt“, CIRS-NRW und ADKA-DokuPIK. Projektbezogen wurde außerdem ein entsprechendes Meldesystem in der Kinder- und Jugendklinik des Universitätsklinikums Erlangen (MF in der Pädiatrie) eingerichtet [41]. Die in diesem Kontext organisierten Berichts‑, Dokumentations- und Lernsysteme erfassen zwar Medikationsfehler, jedoch werden diese derzeit nicht vollständig an die zuständigen Arzneimittelkommissionen bzw. Bundesoberbehörden weitergeleitet.

#### Umgang mit Berichten zu Medikationsfehlern.

Die einzelnen Einrichtungen erreichen unterschiedliche Zielgruppen und üben ihre Aufgaben entweder auf bundesweiter, regionaler oder lokaler Ebene aus. Der Umgang mit den dokumentierten Medikationsfehlern variiert je nach Zielsetzung und den rechtlich verankerten Tätigkeiten der einzelnen Projekte, Initiativen und Institutionen. Durch regelmäßige Veröffentlichungen verfolgen diese Stellen Fortbildungsziele im Themenfeld „Medikationsfehler“ und sensibilisieren damit die unterschiedlichen Fachkreise, insbesondere die Ärzte- und Apothekerschaft sowie Angehörige der Pflegeberufe.

Es gibt zahlreiche Beispiele für überlappende Zuständigkeiten der verschiedenen Stellen: So kann etwa die Verwechslung von Fertigarzneimitteln im Medikamentenschrank einer Krankenhausstationsapotheke dazu führen, dass die Lagerung umorganisiert und krankenhausintern Prozesse angepasst werden. In diesem Fall wird der Vorfall an ein CIRS-System gemeldet. Dieselbe Verwechslungsgefahr kann jedoch auch behördliche Maßnahmen nach sich ziehen, etwa eine Änderung der äußeren Umhüllung der betreffenden Arzneimittel, was durch eine Meldung an ein behördliches Meldesystem bzw. die AkdÄ oder AMK ausgelöst werden kann.

### Medikationsfehler: Anzahl, Art, Setting und betroffene Arzneimittelgruppen

#### Anzahl der Medikationsfehler.

**Abb. 1:**
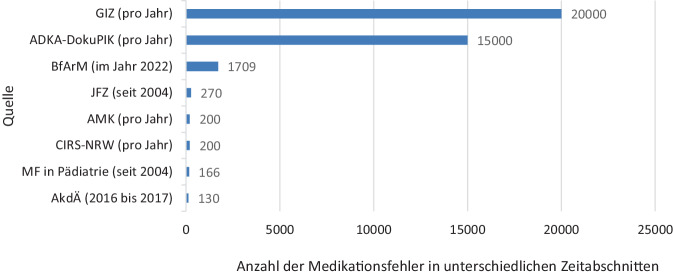
Geschätzte Anzahl der dokumentierten Berichte über Medikationsfehler in den teilnehmenden Institutionen. Die Angaben beziehen sich auf unterschiedliche Zeitabschnitte.* ADKA-DokuPIK* Dokumentation Pharmazeutischer Interventionen im Krankenhaus; *AkdÄ* Arzneimittelkommission der deutschen Ärzteschaft; *AMK* Arzneimittelkommission der Deutschen Apotheker; *BfArM* Bundesinstitut für Arzneimittel und Medizinprodukte; *CIRS-NRW* Critical-Incident-Reporting-System Nordrhein-Westfalen; *GIZ* Giftinformationszentren der Länder; *JFZ* „Jeder-Fehler-zählt“, Fehlerberichts- und Lernsystem für Hausarztpraxen; *MF in Pädiatrie* Meldesystem der Kinder- und Jugendklinik des Universitätsklinikums Erlangen

Die Anzahl der dokumentierten Berichte über Medikationsfehler in den Institutionen variiert sehr stark (Abb. 1). Zu beachten ist, dass sich die Angaben auf unterschiedliche Zeitabschnitte beziehen.

**Abb. 2:**
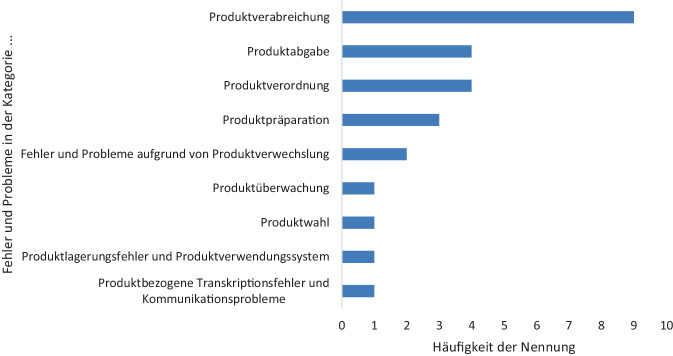
Art der Medikationsfehler nach Häufigkeit der Nennung in Kategorien gemäß MedDRA-Leitfaden Version 24.0

Die GIZ gehen aufgrund von Analysen in Berlin, Erfurt und Zürich davon aus, jährlich bis zu 20.000 Beratungen durchzuführen, die einen Bezug zu Medikationsfehlern aufweisen. Sie heben sich mit dieser Angabe deutlich von den weiteren Beteiligten ab. Beim BfArM wurden im Jahr 2022 mehr als 1700 Berichte erfasst, die einen AMTS-Bezug aufwiesen, und der AMK werden jährlich etwa 200 Medikationsfehler gemeldet, Tendenz steigend [42]. Der AkdÄ wurden im Zusammenhang mit dem „Projekt zur Erfassung und Bewertung von Medikationsfehlern“ während der 2‑jährigen Laufzeit 130 eindeutig als Medikationsfehler kodierbare Fälle mitgeteilt. Über das ADKA-DokuPIK-Portal werden jährlich etwa 15.000 Medikationsfehler erfasst. Im Vergleich dazu dokumentiert CIRS-NRW pro Jahr ca. 200 Medikationsfehler. Die Initiative „Jeder-Fehler-zählt“ sowie das Projekt zum Meldesystem der Kinder- und Jugendklinik des Universitätsklinikums Erlangen (MF in der Pädiatrie) haben seit 2004 jeweils mehr als 250 bzw. über 150 Medikationsfehler erfasst [41].

Die Anzahl der dokumentierten Medikationsfehler unterscheidet sich je nach zugrunde liegendem Auftrag bzw. den Zielen der zuständigen Stellen bzw. Systeme. So ist es ein Unterschied, ob die Arzneimittelkommissionen – AkdÄ und AMK – Meldungen im Rahmen des Spontanmeldewesens entgegennehmen oder ob gezielt Medikationsfehler aufgrund von speziellen Schulungsmaßnahmen oder Interventionen identifiziert und an die Berichtssysteme übermittelt werden, z. B. an ADKA-DokuPIK. Spezielle Schulungen können zu einer erhöhten Sensibilität für Medikationsfehler führen. Dieser Effekt wurde auch nach Fortbildung der niedergelassenen Ärzteschaft in Qualitätszirkeln und im Zusammenhang mit der Einführung eines spezifisch für Kinder und Jugendliche erstellten Arzneimittelinformationssystems „Kinderformularium“ beobachtet [31].

#### Art der Medikationsfehler.

Die von den Teilnehmenden angegebenen Arten von dokumentierten Medikationsfehlern wurden Kategorien zugeordnet, die sich an den High Level Terms (HLT) des MedDRA-Leitfadens, Version 24.0, deutsche Fassung, orientieren, und nach Häufigkeit der Nennung ausgewertet ([43]; Abb. 2). Diese Einteilung umfasst die Stadien des Medikationsprozesses gemäß dem „Good practice guide on recording, coding, reporting and assessment of medication errors“ (EMA/762563/2014): Verordnung, Lagerung, Abgabe, Vorbereitung zur Verabreichung und Verabreichung [44]. Am häufigsten wurden mit 9 Nennungen Fehler und Probleme bei der Verabreichung von Arzneimitteln berichtet. Fast die Hälfte der Beteiligten gab Fehler und Probleme bei der Verordnung sowie bei der Abgabe an. Mit 3 bzw. 2 Nennungen waren auch Fehler im Zusammenhang mit der Vorbereitung der Arzneimittel sowie Verwechslungen von Bedeutung. Zudem wurden jeweils einmal Fehler im Zusammenhang mit Kommunikationsproblemen, der Lagerung, der Auswahl der Arzneimittel und dem Monitoring genannt. Insgesamt bestätigt sich: Medikationsfehler können in jedem Schritt des Medikationsprozesses auftreten.

#### Settings der Medikationsfehler.

Die Befragten machten auch Angaben über die berichteten Versorgungssituationen, in denen Medikationsfehler wahrgenommen werden. Medikationsfehler werden gleichermaßen im ambulanten und im stationären Setting berichtet. Basierend auf der Abfrage, wurde das Krankenhaus mit 9 Nennungen am häufigsten erwähnt, gefolgt von der Arztpraxis, Pflegeeinrichtungen sowie der häuslich-privaten Umgebung mit jeweils 3 Nennungen. Die öffentliche Apotheke, Krankenhausapotheke sowie die Notfallversorgung wurden jeweils 2‑mal als Versorgungssituation für das Auftreten von Medikationsfehlern erwähnt. Somit wird deutlich: Alle am Medikationsprozess Beteiligten können Fehler machen, jedoch lassen sich Fehler auch bei jedem Schritt des Prozesses vermeiden.

#### Betroffene Arzneimittelgruppen.

Insgesamt wurden von den Befragten 13 therapeutische Hauptgruppen nach Anatomisch-Therapeutisch-Chemischer-(ATC-)Klassifikation im Kontext mit Berichten über Medikationsfehler aufgeführt [45]. Am häufigsten wurde die Hauptgruppe N02 Analgetika genannt, etwa Acetylsalicylsäure‑, Ibuprofen- und Metamizol-haltige Arzneimittel. Eine wichtige Rolle spielen zudem die Arzneimittelgruppen B01 antithrombotische Mittel, N05 Psycholeptika sowie J01 Antibiotika zur systemischen Anwendung. Beispielhaft für die therapeutische Hauptgruppe B01 antithrombotische Mittel wurden Apixaban‑, Dabigatran- sowie Phenprocoumon- und Rivaroxaban-haltige Arzneimittel aufgeführt.

### Herausforderungen und förderliche Faktoren im Meldewesen

Die Befragten äußerten sich in der Erhebung zu möglichen Herausforderungen und förderliche Faktoren im Hinblick auf das Zusammenwirken der einzelnen Projekte, Initiativen und Institutionen im Meldewesen. Als Hemmnisse und Herausforderungen wurden insbesondere fehlende personelle, finanzielle und zeitliche Ressourcen genannt. Zudem wurden mangelnde Kenntnisse über die Meldewege, die akute Mehrbelastung durch die SARS-CoV-2-Pandemie und haftungsrechtliche Befürchtungen als wesentliche Barrieren für das Melden von Medikationsfehlern identifiziert. Diese Aspekte könnten möglicherweise dazu beitragen, dass Medikationsfehler seltener gemeldet werden (Underreporting; [46]).

Daneben wurden auch Probleme bei der Identifikation von Medikationsfehlern im Zusammenhang mit Berichten über Arzneimittelnebenwirkungen diskutiert. Es werden etwa nicht immer die konkreten Umstände bzw. Ursachen beschrieben, die Medikationsfehler begünstigen. Zudem werden Missbrauch und Fehlgebrauch von Medikamenten gemeldet, die nicht direkt mit der regulatorischen Definition eines Medikationsfehlers übereinstimmen. Für die Analyse und Bewertung von Meldungen über Medikationsfehler fehlen oftmals relevante Informationen, wie die vollständige Bezeichnung des betroffenen Arzneimittels oder der konkrete Schritt im Medikationsprozess. Insgesamt erfolgt die Erfassung von Fehlerszenarien in den verschiedenen Projekten, Initiativen und Institutionen uneinheitlich.

Eine besondere Rolle spielen in diesem Zusammenhang die Berichte über *potenzielle Medikationsfehler*. Darunter werden Umstände oder Informationen verstanden, die zu einem Medikationsfehler führen könnten, wobei eine Patientin bzw. ein Patient betroffen sein kann oder auch nicht [44]. Die Meldungen über potenzielle Medikationsfehler werden im Gegensatz zu Medikationsfehlern mit Nebenwirkung nicht an die europäische Datenbank EudraVigilance weitergeleitet. Folglich stehen diese Berichte auch nicht für entsprechende Auswertungen auf europäischer Ebene zur Verfügung – sind aber ein ganz wichtiger Bestandteil im Risikomanagement und haben ein präventives Potenzial.

Die Rückmeldungen aus dem Teilnehmerkreis des Workshops deuten darauf hin, dass insbesondere die Etablierung einer positiven Fehlerkultur, die Möglichkeit zur anonymen Meldung und ein vereinfachter Meldeprozess das Melden von Medikationsfehlern begünstigen. Neben Aufwandsentschädigungen wird auch ein gutes Vertrauensverhältnis innerhalb des therapeutischen Teams als wesentlicher Faktor für die Effektivität und Akzeptanz von Meldesystemen betrachtet.

### Zusammenwirken und Einrichtung von Schnittstellen

**Abb. 3:**
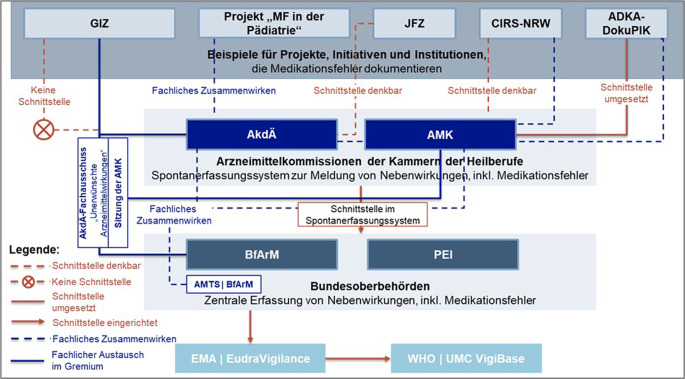
Erfassung von Nebenwirkungen und Medikationsfehlern: Zusammenwirken der unterschiedlichen Projekte, Initiativen und Institutionen mit den Arzneimittelkommissionen – AkdÄ und AMK – sowie den Bundesoberbehörden im Rahmen des fachlichen Austausches bzw. der Vernetzung über Schnittstellen im Spontanmeldesystem. *ADKA-DokuPIK* Dokumentation Pharmazeutischer Interventionen im Krankenhaus; *AkdÄ* Arzneimittelkommission der deutschen Ärzteschaft; *AMK* Arzneimittelkommission der Deutschen Apotheker; *AMTS* Arzneimitteltherapiesicherheit; *BfArM* Bundesinstitut für Arzneimittel und Medizinprodukte; *CIRS-NRW* Critical-Incident-Reporting-System Nordrhein-Westfalen; *EMA EudraVigilance* Europäische Arzneimittel-Agentur, Informationsnetzwerk „European Union Drug Regulating Authorities Pharmacovigilance“; *GIZ* Giftinformationszentren der Länder; *JFZ* „Jeder-Fehler-zählt“, Fehlerberichts- und Lernsystem für Hausarztpraxen; MF in der Pädiatrie: Meldesystem der Kinder- und Jugendklinik des Universitätsklinikums Erlangen; *PEI* Paul-Ehrlich-Institut; *UMC VigiBase* Uppsala Monitoring Centre, globale Datenbank für Individual Case Safety Reports (ICSR); *WHO* Weltgesundheitsorganisation

In Abb. 3 sind die Rückmeldungen zum Zusammenwirken der teilnehmenden Projekte, Initiativen, Institutionen, Arzneimittelkommissionen und Behörden auf dem Gebiet „Medikationsfehler“ grafisch dargestellt. Der fachliche Austausch sowie die systematische Weiterleitung von Berichten über Medikationsfehler sind auf unterschiedlichen Ebenen zwischen den beteiligten Akteurinnen und Akteuren möglich.

Die Arzneimittelkommissionen der Kammern der Heilberufe – AkdÄ und AMK – haben im Rahmen des Spontanmeldesystems Schnittstellen zum BfArM und PEI eingerichtet. Über diesen Weg werden Berichte über Medikationsfehler systematisch an die beiden zuständigen Bundesoberbehörden weitergeleitet. Beim BfArM wurde zudem die Arbeitsgruppe „AMTS“ eingerichtet, um die fachliche Zusammenarbeit zwischen AkdÄ, AMK und BfArM zu koordinieren. Darüber hinaus findet halbjährlich die Routinesitzung nach § 63 AMG im BfArM statt, in der die Auswertung eingegangener Meldungen über Nebenwirkungen und Arzneimittelrisiken erörtert wird. Bei der AkdÄ existiert zudem der Fachausschuss „Unerwünschte Arzneimittelwirkungen“, in dem Expertinnen und Experten der GIZ, der AMK, des BfArM und PEI regelmäßig in den fachlichen Austausch treten – auch auf dem Gebiet der AMTS. Auch die AMK widmet sich in ihren halbjährlichen Sitzungen zentralen Fragen der AMTS, an denen u. a. die AkdÄ, die ADKA und das BfArM teilnehmen.

Weiterhin ist die Zusammenarbeit auf Fachebene zwischen AMK und CIRS-NRW bzw. ADKA-DokuPIK sowie zwischen AkdÄ und dem Projekt „KIDSafe“ etabliert. Die Übermittlung von Berichten über Medikationsfehler aus anderen Systemen an die AkdÄ bzw. AMK ist auch über die Einrichtung von Schnittstellen denkbar. Eine solche Schnittstelle ist zwischen ADKA-DokuPIK und AMK bereits umgesetzt.

Die GIZ, das Projekt JFZ sowie CIRS-NRW leiten Berichte über Medikationsfehler nicht systematisch an die Bundesoberbehörden oder Arzneimittelkommissionen weiter. Grundsätzlich wären auch hier Schnittstellen zwischen der JFZ-Initiative und der AkdÄ bzw. zwischen CIRS-NRW und der AMK denkbar. Die GIZ stehen vor Herausforderungen bei der systematischen Meldung von Fällen, da föderale Strukturen in diesem Bereich eine einheitliche Datenerhebung verhindern. Zudem erschwert der bestehende Ressourcenmangel die Einrichtung einer zentralen Schnittstelle, die eine strukturierte Erfassung und Meldung relevanter Fälle ermöglichen könnte.

## Einordnung der Ergebnisse und Schlussfolgerungen

Der vorliegende Bericht über die Ergebnisse des Workshops mit Expertendiskussion sowie die ergänzende Erhebung bieten zwar einen umfassenden Überblick über die unterschiedlichen Projekte, Initiativen und Institutionen in Deutschland, bei denen Meldungen zu Medikationsfehlern eingehen. Gleichwohl bedarf es einer Einordnung der Resultate. Das 2‑stufige Vorgehen, bestehend aus einer Expertendiskussion im Workshop und einer ergänzenden Erhebung im Teilnehmerkreis ermöglicht lediglich die Feststellung von Tendenzen [47]. Die Ergebnisse sind daher nicht quantitativ beurteilbar und hinsichtlich ihrer Generalisierbarkeit eingeschränkt. Sie spiegeln den Erfahrungshorizont der Beteiligten wider, wodurch komplexe Zusammenhänge im Umgang mit Medikationsfehlern an den einzelnen Stellen besser verstanden und erklärt werden können. Eine mögliche Einschränkung dieser Zusammenschau besteht weiterhin darin, dass nicht alle Projekte, Initiativen und Institutionen aus Deutschland mit Bezug zu Medikationsfehlern vollumfänglich in diese Auswertung einbezogen werden konnten.

In Deutschland werden an vielen Stellen Medikationsfehler gemeldet, die jedoch in verschiedenen Strukturen und unter unterschiedlichen Gesichtspunkten erfasst werden. Ein Austausch der einzelnen Systeme, z. B. über gemeinsame Schnittstellen, zur Ableitung von übergreifenden Erkenntnissen existiert derzeit nicht.

Ein besonderes Augenmerk sollte auf die Erfassung *potenzieller Medikationsfehler* gelegt werden, die bisher nicht regelhaft in die europäische EudraVigilance-Datenbank eingespeist werden. Potenzielle Medikationsfehler haben bei guter Dokumentationsqualität ihren eigenen Stellenwert und sind für eine Gesamtbetrachtung der fehlerbegleitenden Umstände im Medikationsprozess notwendig. Auf dieser Basis könnten ggf. behördliche Maßnahmen im Sinne des vorbeugenden Patientenschutzes eingeleitet werden.

Im Hinblick auf die Meldung von Medikationsfehlern und deren anschließende Erfassung ist es aus behördlicher Sicht wünschenswert, Szenarien wie absichtliche Überdosierung, Fehlgebrauch, Missbrauch oder berufliche bzw. versehentliche Exposition deutlich von Medikationsfehlern abzugrenzen. Zudem ist es für die Dokumentation wichtig, die aufgetretenen Fehler im Medikationsprozess detailliert zu beschreiben, um die ursächlichen Zusammenhänge präziser zu erfassen. Dieser Aspekt gewinnt insbesondere vor dem Hintergrund der Digitalisierung im Gesundheitswesen an Bedeutung, da etwa fehlerhaft ausgestellte elektronische Rezepte oder Medikationspläne potenziell zu Medikationsfehlern führen können [48].

Die Ergebnisse verdeutlichen, dass von allen Beteiligten in jedem Schritt im Medikationsprozess und in allen Versorgungssituationen Fehler verursacht werden können, jedoch auch Vermeidungspotenzial besteht. Eine positive Fehlerkultur und ein gutes Vertrauensverhältnis innerhalb eines interprofessionellen therapeutischen Teams können dazu beitragen, die Bereitschaft zum Melden von Medikationsfehlern zu erhöhen. Dadurch besteht die Möglichkeit, gemeinsam aus Fehlern zu lernen, präventive Maßnahmen abzuleiten und zukünftig Fehler zu verhindern. Zudem könnten gezielte regulatorische Maßnahmen getroffen werden, um das Auftreten von Medikationsfehlern nachhaltig zu reduzieren. Eine zentrale Vernetzung könnte dazu beitragen, personelle, zeitliche und finanzielle Ressourcen effizienter zu nutzen und gezielt zu bündeln. Die Verstetigung des Fachaustausches unter den Expertinnen und Experten auf dem Gebiet der Medikationsfehler sollte durch den Aktionsplan AMTS des BMG unterstützt werden.

### Infobox Definitionen

**Nebenwirkungen **– sind schädlich und unbeabsichtigt …

„Nebenwirkungen sind schädliche und unbeabsichtigte Reaktionen auf das Arzneimittel. Schwerwiegende Nebenwirkungen sind Nebenwirkungen, die tödlich oder lebensbedrohend sind, eine stationäre Behandlung oder Verlängerung einer stationären Behandlung erforderlich machen, zu bleibender oder schwerwiegender Behinderung, Invalidität, kongenitalen Anomalien oder Geburtsfehlern führen. Unerwartete Nebenwirkungen sind Nebenwirkungen, deren Art, Ausmaß oder Ergebnis von der Fachinformation des Arzneimittels abweichen“ [6].

**Medikationsfehler** – sind vermeidbar …

„Ein Medikationsfehler ist ein Abweichen von dem für den Patienten optimalen Medikationsprozess, das zu einer grundsätzlich vermeidbaren Schädigung des Patienten führt oder führen könnte“ [7].

**Medikationsfehler** – geschehen unbeabsichtigt …

„Ein Medikationsfehler ist ein unbeabsichtigtes Versagen im Medikationsprozess, das zu Schaden beim Patienten führt oder führen könnte“ (dt. Übersetzung aus [44]).

**Arzneimitteltherapiesicherheit (AMTS) **– betrachtet den gesamten Prozess …

„AMTS ist die Gesamtheit der Maßnahmen zur Gewährleistung eines optimalen Medikationsprozesses mit dem Ziel, Medikationsfehler und damit vermeidbare Risiken für den Patienten bei der Arzneimitteltherapie zu verringern“ [7].
